# Selective suppression of the JNK-MMP2/9 signal pathway by tetramethylpyrazine attenuates neuropathic pain in rats

**DOI:** 10.1186/s12974-017-0947-x

**Published:** 2017-08-31

**Authors:** Lai Jiang, Cai-Long Pan, Chao-Yu Wang, Bing-Qian Liu, Yuan Han, Liang Hu, Lei Liu, Yang Yang, Jun-Wei Qu, Wen-Tao Liu

**Affiliations:** 1 Department of Pharmacology, Jiangsu Key Laboratory of Neurodegeneration, Nanjing Medical University, 140 Han-Zhong Road, Nanjing, 210029 China; 2 Department of Ophthalmology, the First Affiliated Hospital with Nanjing Medical University, Nanjing, 210029 China; 3 Jiangsu Province Key Laboratory of Anesthesiology, Xuzhou Medical College, Xuzhou, 221000 China; 4 Department of pain, Shandong Qianfoshan Hospital, Shandong, 250014 China; 5 Department of Gynecologic Oncology, Jiangsu Institute of Cancer Research, Jiangsu Cancer Hospital, Nanjing Medical University Affiliated Cancer Hospital, Nanjing, Jiangsu 210009 China

## Abstract

**Background:**

Activated astrocytes release matrix metalloproteinase-2/9 (MMP-2/9) to induce central sensitization and maintain neuropathic pain. However, the mechanisms involved in the activation of MMP-2/9 on astrocytes during pain remain poorly understood. Meanwhile, there is a lack of effective treatment to inhibit the activation of MMP-2/9 on astrocytes. In this study, we aim to investigate the effect of tetramethylpyrazine (TMP), a natural compound with analgesic effects but unknown mechanisms, on MMP-2/9 in neuropathic pain.

**Methods:**

The nociception was assessed by measuring the incidence of foot withdrawal in response to mechanical indentation in rats (*n* = 6). Cell signaling was assayed using western blotting (*n* = 6) and immunohistochemistry (*n* = 5). The astrocyte cell line C8-D1A was cultured to investigate the in vitro effects.

**Results:**

TMP significantly attenuated the maintenance of chronic constrictive injury (CCI)-induced neuropathic pain, inhibited the activation of astrocytes, and decreased the expression of MMP-2/9. Furthermore, our results indicated that TMP could selectively suppress JNK activity but had no notable effects on ERK and p38*.* Our study also revealed that the effect of TMP may be dependent on the inhibition of TAK1.

**Conclusions:**

Inhibition of astrocyte activation in the spinal cord by tetramethylpyrazine may have utility in the treatment of CCI-induced neuroinflammation, and our results further implicate JNK-MMP-2/9 as a novel target for the attenuation of neuropathic pain.

**Electronic supplementary material:**

The online version of this article (10.1186/s12974-017-0947-x) contains supplementary material, which is available to authorized users.

## Background

Neuropathic pain is defined as pain caused by a lesion or disease of the somatosensory nervous system [1]. Management of neuropathic pain remains a clinical challenge: current treatments for chronic pain have been greatly limited by an incomplete understanding of its pathogenesis. The continuous search for a safer and more effective compound is urgently needed.

Convincing evidence shows that nerve injury induces the profound activation of glial cells, including microglia and astrocytes, in the spinal cord [2]. Microglia and astrocytes both play important roles in neuropathic pain. Activation of spinal microglial cell occurs in the early phase after injury and is critical in the induction of neuropathic pain [3]. Compared with the microglial response, astrocytes are activated in the late phase after injury and are thought to be important in the maintenance of neuropathic pain [3]. Astrocyte proliferation begins relatively late and progresses slowly but is sustained for a longer period (more than 5 months) [4]. Further studies indicate that intrathecal injection of the astrocyte inhibitor fluoroacetate could significantly alleviate behaviors associated with neuropathic pain in animal models [5].

Activated astrocytes produce numerous proinflammatory cytokines (such as IL-1β and TNF-α) [6], chemokines (such as CX3CL1 (fractalkine) and CCL2 (MCP-1)) [7], and algogenic substance (such as MMP-2/9) [8] to facilitate central sensitization [9]. Among all the pain-related substrates, MMP-2/9 has attracted increasing attention for neuropathic pain [10]. MMP-9 contributes to the early stage of neuropathic pain, while MMP-2 maintains neuropathic pain [11]. On the one hand, MMP-2/9 contributes to the maturation of IL-1β, which increases NMDA receptor phosphorylation via a PKCγ-dependent manner [10]. On the other hand, a previous study by our group showed that spinal MMP-9 could enhance the activation of *N*-methyl-D-aspartate (NMDA) receptors via the integrin-β1-mediated signal cascade [12]. Hence, there is an urgent need for a safe and effective astrocyte and MMP-2/9 inhibitors that can be used in the clinic for the treatment of neuropathic pain.

Based on the information mentioned above, preventing the activation of astrocytes and MMP-2/9 is becoming an attractive target for suppressing neuropathic pain. Our group is focused on searching for an “old” drug, which, historically, has been clinically effective and safe, to manage neuropathic pain via inhibition of MMP-2/9. After screening a variety of compounds and based on our group’s preliminary data, we focused on tetramethylpyrazine (TMP), the bioactive component extracted from chuanxiong (*Ligusticum chuanxiong hort*). The reason we choose TMP as a research target is based on the following considerations: First, chuanxiong, a Chinese traditional medicine, has been used for treating chronic pain for more than 100 years [13]. Second, TMP can significantly decrease the migration and proliferation of glioma cells in rats [14]. Additionally, it is widely known that high MMP-2/9 expression is a key characteristic of gliomas, which could significantly accelerate remodeling [13], development, and angiogenesis [15]. Third, TMP has been widely used for three decades in clinical treatments, including ischemic and cerebral infarction diseases of the central nervous system to suppress neuroinflammation [14]. Therefore, we hypothesized that TMP might suppress chronic constrictive injury (CCI)-induced MMP-2/9 from astrocytes. In the current study, we examined whether TMP inhibits MMP-2/9 in astrocytes.

## Methods

### Ethics statement

All procedures were strictly performed in accordance with the regulations of the ethics committee of the International Association for the Study of Pain and the Guide for the Care and Use of Laboratory Animals (The Ministry of Science and Technology of China, 2006). All animal experiments were approved by Nanjing Medical University Animal Care and Use Committee and were designed to minimize the suffering and the number of animals used.

### Animals and neuropathic pain model

Adult male Sprague-Dawley rats (180–200 g wt.) were provided by the Experimental Animal Center at Nanjing Medical University, Nanjing, China. Animals were housed five to six per cage under pathogen-free conditions with soft bedding under controlled temperature (22 ± 2 °C) and a 12-h light/dark cycle (lights on at 8:00 a.m.). Behavioral testing was performed during the light cycle (between 9:00 a.m. and 5:00 p.m.). The animals were allowed to acclimate to these conditions for at least 2 days before starting experiments. Animals were randomly divided into groups (*n* = 6). The sample size was designed on prior experience and to be limited to the minimal as scientifically justified). For each group of experiments, the animals were matched by age and body weight. All surgeries were done under anesthesia induced by chloral hydrate (300 mg/kg, i.p.). Peripheral nerve injury was imitated by the model of chronic constriction injury (CCI) of the sciatic nerve. In brief, the left common sciatic nerve of each rat was exposed at the mid-thigh level. Proximal to the sciatic nerve’s trifurcation, approximately 7 mm of nerve was separated from adhering tissue and four ligatures (4–0 chronic gut) were tied loosely around it with about 1 mm between ligatures. After surgery, the skin layers and muscle were sutured, and the surgery area was sterilized with iodine.

### Drugs and reagents

Tetramethylpyrazine was purchased from Zelang Pharmaceutical Co. Ltd. (Nanjing, China). The purity of tetramethylpyrazine was more than 99%. TMP **was** dissolved in sterile saline (0.9%), the allocation of different concentrations of TMP solution, **and** the concentration of TMP was 1, 3, **and** 9 mg/ml. Solvent**-**treated controls were injected with sterile saline. Fetal bovine serum (FBS) and other cell culture media and supplements were purchased from Hyclone (USA). SP600125 was purchased from beyotime biotechnology**, and** 5Z-7-oxozeaenol was purchased from Millipore. Anti-glyceraldehyde 3-phosphatedehydrogenase (GAPDH) was from Sigma. Anti-Phospho-p38 MAPK was from Cell Signaling Technology (Beverly, MA, USA). Anti-ionized calcium-binding adapter molecule 1 (IBA-1), anti-JNK, anti-MMP9, and anti-MMP2 were from Abcam (USA). Anti-phosphorylated *N*-methyl-D-aspartate receptor (NMDAR) NR1 subunit (Ser896) was from Cell Signaling Technology (Beverly, MA, USA). Anti-phospho-PKC (pan) (gamma Thr514) Antibody was from Cell Signaling Technology (Beverly, MA, USA). Anti-GFAP, anti-p44/42 MAPK (Erk 1/2), and anti-p38 MAPK were from Cell Signaling Technology (Beverly, MA, USA). Anti-Phospho-p44/42 MAPK (Erk1/2) was from Cell Signaling Technology (Beverly, MA, USA). Anti-phosphoSAPK/JNK was from Cell Signaling Technology (Beverly, MA, USA). TAK1 was from Cell Signaling Technology (Beverly, MA, USA). Secondary antibodies were from Cell Signaling Technology (Beverly, MA, USA). All other chemicals were purchased from Sigma.

### Cell culture

Astrocyte C8-D1A cells were incubated under humidified 5% CO2 and 95% O2 at 37 °C in Dulbecco’s modified Eagle’s medium (DMEM; Invitrogen, USA) containing 10% FBS and 1% streptomycin and penicillin (Invitrogen). Twenty-four hours before experimentation, the culture media was replaced by 0.5% FBS high-glucose DMEM. Then, the cells were stimulated with IL-1β (20 ng/ml) for 30 min with or without tetramethylpyrazine (0.5, 5, and 50 μM).

### Intrathecal injection procedure

To perform intrathecal (i.t.) injections, the rat was placed in a prone position and the midpoint between the tips of the iliac crest was located. A Hamilton syringe with 30-gauge needle was inserted into the subarachnoid space of the spinal cord between the L5 and L6 spinous processes. Proper intrathecal injection was systemically confirmed by observation of a tail flick. Intrathecal injection did not affect baseline responses, compared with latencies recorded before injection.

### Gelatin zymography

Animals were anesthetized deeply with chloral hydrate (300 mg/kg, i.p.), and spinal cord segments (L1–L6) were rapidly dissected and homogenized in 1% NP40 lysis. 300–500 μg of protein per lane was loaded into the wells of precast gels (8% polyacrylamide gels containing 0.1% gelatin). After electrophoresis, each gel was incubated with 50 ml of developing buffer for 48 h (37.5 °C) in shaking bath. Then, the gels were stained with coomassie brilliant blue (1%, with 10% acetic acid, 10% isopropyl alcohol, diluted with dd H2O).

### Western blotting

The entail spinal cord segments at L1–L6 were rapidly collected at 4 h after the last drug administration. The protein concentrations were determined by BCA Protein Assay (Thermo Fisher, Waltham, MA, USA), and 40–80 μg of proteins were loaded and separated by SDS-PAGE and electrophoretically transferred onto polyvinylidene fluoride membranes (Millipore Corp., Bedford, MA). The membranes were blocked with 5% bovine serum albumin for 1 h at room temperature, probed with antibodies overnight at 4 °C with the primary antibodies and then incubated with HRP-coupled secondary antibodies. The primary antibodies used included IL-1β (1:300), p-NR1(1:1000), p-PKCγ (1:1000), p-p38 (1:1000), p-JNK(1:1000), p-ERK (1:1000), p-TAK1(1:1000). For loading control, the blots were probed with antibody for GAPDH (1:8000). The filters were then developed by enhanced chemiluminescence reagents (Perkinelmer) with secondary antibodies (Chemicon). Data were analyzed with the Molecular Imager (Gel DocTM XR, 170–8170) and the associated software Quantity One-4.6.5(Bio-Rad Laboratories, Berkeley, CA).

### Behavioral analysis

Animals were habituated to the testing environment daily for at least 2 days before baseline testing. Mechanical sensitivity was detected by von Frey Hairs (Woodland Hills, LA, USA) test. Animals were placed in boxes set on an elevated metal mesh floor and were allowed 30 min for habituation before testing. The plantar surface of each hind paw was stimulated with a series of von Frey hairs with logarithmically incrementing stiffness perpendicularly to the plantar surface. Each rat was tested for three times, and the average of the threshold was measured.

### Immunofluorescence

After deep anesthesia by intraperitoneal injection of chloral hydrate (300 mg/kg), the animal was perfused transcardially with normal saline followed by 4% paraformaldehyde in 0.1 M PB, pH 7.4, each for 20 min. Then, L4 and/or L5 lumbar segment was dissected out and post-fixed in 4% paraformaldehyde. The embedded blocks were sectioned as 25 μm thick. Sections from each group (five mice in each group) were incubated with goat antibodies for GFAP (1:200), p-JNK(1:100). Then, the free-floating sections were washed with PBS and incubated with the secondary antibody for 2 h. After washing out three times with PBS, the samples were studied under an immunofluorescence microscope (Zeiss AX10, Germany) for morphologic details of the immunofluorescence staining. Examination was blindly carried out. Images were randomly coded, and the fluorescence intensities were analyzed by Image Pro Plus 6.0 software (Media Cybernetics Inc. Rockville, MD, USA). The average green fluorescence intensity of each pixel was normalized to the background intensity in the same image.

### RT-PCR

Samples (spinal cord segments at L1-L6) were homogenized in Trizol reagent (Invitrogen Life Technologies, Carlsbad, CA, USA), and total RNA was treated by DNaseI and subjected to quantitative PCR, which was performed with ABI Prism 7300 sequence detection system (Applied Biosystems, Foster City, CA, USA) using SYBR Green I dye. The sense and antisense primers used for the analysis of rat MMP-9, MMP-2, and GAPDH expression were as follows: MMP-9: 5′-TCGAAGGCGACCTCAAGTG-3′ and 5′-TTCGGTGTAGCTTTGGATCCA-3′, MMP-2: 5′-ACCGTCGCCCATCATCAA-3′ and 5′-TTGCACTGCCAACTCTTTGT CT-3′, and GAPDH: 5′-ATGACTCTACCCACGGCAAG-3′ and 5′-CTGGAAGATGGT GATGGGTT-3′.

### Statistical analyses

SPSS Rel 15 (SPSS Inc., Chicago, IL, USA) was used to conduct all the statistical analyses. Alteration of expression of the proteins detected and the behavioral responses were tested with one-way ANOVA, and the differences in latency over time among groups were tested with two-way ANOVA. Bonferroni post hoc tests were conducted for all ANOVA models. Results are expressed as mean ± SD of three independent experiments. Results described as significant are based on a criterion of *P* < 0.05.

## Results

### TMP treatment attenuated CCI-induced neuropathic pain

**Fig. 1:**
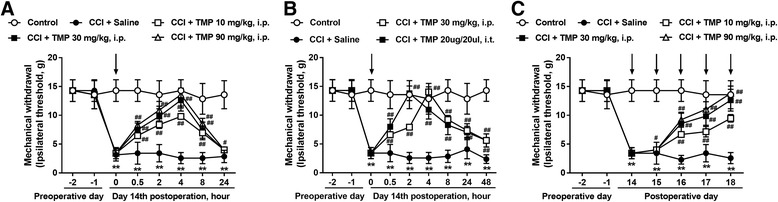
TMP treatment attenuated CCI-induced neuropathic pain. **a** Single administration of TMP (10, 30, and 90 mg/kg, i.p.) significantly attenuated CCI-induced mechanical allodynia. **b** Intrathecal injection of TMP (20 μg/20 μl) attenuated CCI-induced mechanical allodynia. **c** Consecutive administration of TMP (10, 30, and 90 mg/kg, i.p.) for 5 days significantly attenuated CCI-induced mechanical allodynia. Drug administration is indicated by the *arrows* (*n* = 6 each group). Two-way ANOVA revealed a significant difference at *P < 0.05 and **P < 0.01 vs. control; and ^#^ *P* < 0.05 and ^##^ *P* < 0.01 vs. CCI + saline group (Bonferroni post hoc tests)

First, we measured the mechanical threshold of CCI-treated rats using the von Frey test. Fourteen days after the CCI surgery, the mechanical threshold was markedly decreased in CCI-treated rats. A single dosage of TMP (10, 30, and 90 mg/kg, i.p.) was given intraperitoneally to CCI rats, and the mechanical withdrawal threshold was greatly increased (Fig. 1a).

Furthermore, the action of TMP was studied by continuous administration for 5 days from the 14 days after the CCI surgery. In the CCI group, the rats’ mechanical threshold was reduced. After continuous administration of TMP (10, 30, and 90 mg/kg, i.p.) for 5 days, the mechanical threshold was elevated (Fig. 1c).

To provide additional evidence, TMP was administered intrathecally to the CCI-treated rats. The mechanical allodynia was notably ameliorated by a single dose of TMP (Fig. 1b). These data partially address that a local administration targeting spinal cord mechanisms would induce similar effects to those observed after systemic administration.

### TMP could significantly inhibit CCI-induced astrocyte activation

**Fig. 2:**
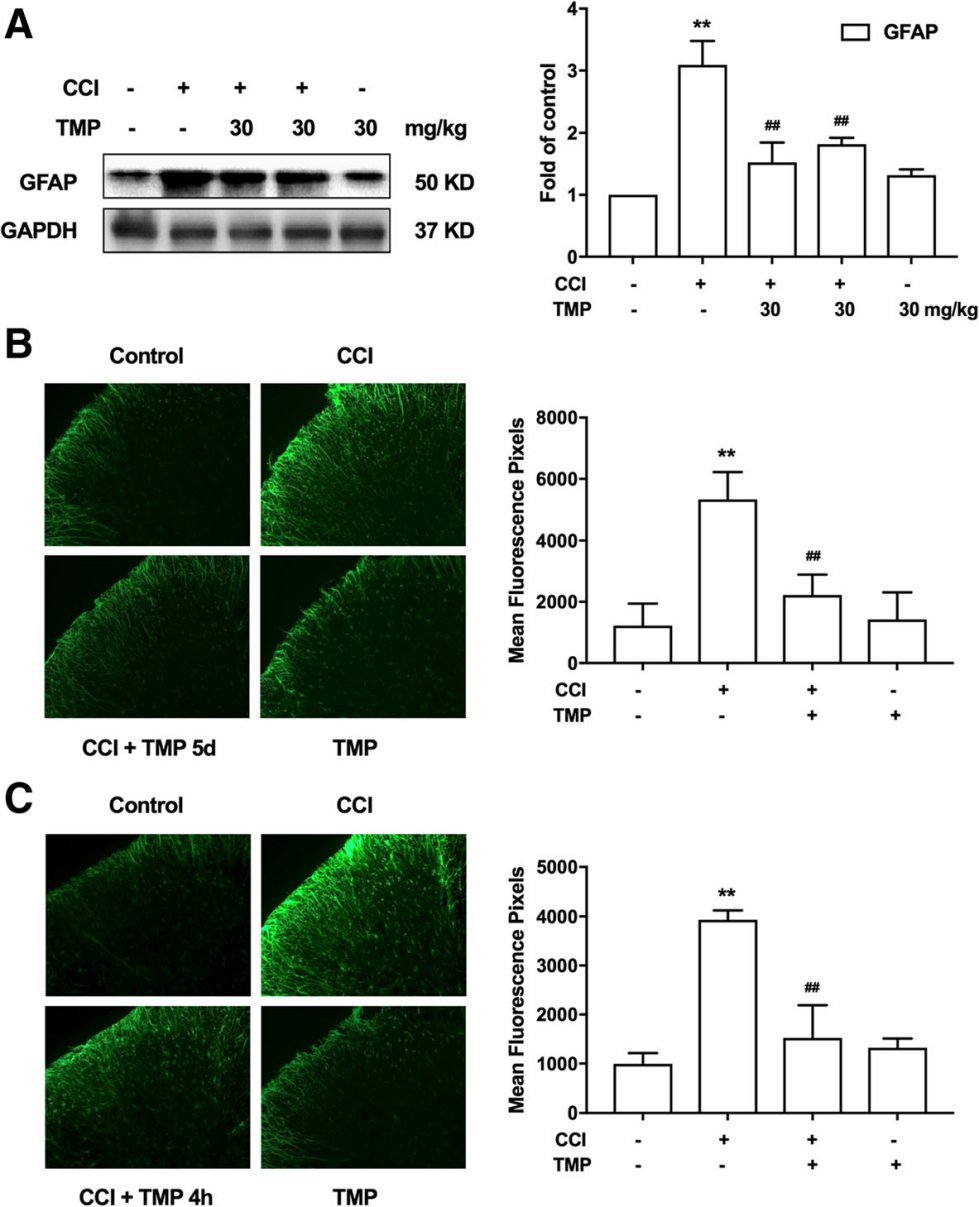
TMP significantly inhibited CCI-induced activation of astrocytes. **a** Single administration (the third band) and consecutive administration (the fourth band) of TMP (30 mg/kg, i.p.) significantly suppressed CCI-induced expression of GFAP in the spinal cord. **b** Consecutive administration of TMP (30 mg/kg, i.p.) significantly suppressed CCI-induced expression of GFAP in the spinal cord. TMP (30 mg/kg, i.p.) was consecutively administered daily from days 14 to 18 after the CCI operation. The lumbar spines (L4–L6) were collected and analyzed at 4 h after the last drug administration. **c** Single administration of TMP (30 mg/kg, i.p.) significantly suppressed CCI-induced expression of GFAP in the spinal cord. The lumbar spines (L4–L6) were collected and analyzed at 4 h after TMP administration. Confocal images and immunofluorescence analysis data showing GFAP in the dorsal horns. Quantification of immunofluorescence was represented as the mean fluorescent pixels in the superficial dorsal horns. One-way ANOVA revealed a significant difference at *P < 0.05 and **P < 0.01 vs. control; and ^#^ *P* < 0.05 and ^##^ *P* < 0.01 vs. CCI group

Glial cell activation and the crosstalk between glia and neurons enhance central sensitization. We evaluated whether TMP affects the activation of glia induced by the CCI operation. Our results showed that TMP (30 mg/kg, i.p.) could significantly suppress the upregulated astrocyte marker GFAP in the spinal cord after the CCI operation (Fig. 2a). However, TMP did not show a notable influence on the microglia marker IBA1 (Additional file 1: Figure S2). Further, immunofluorescence of GFAP in the dorsal horn of CCI-treated rats showed the activation of astrocytes. Notably, this activation was alleviated by TMP (Fig. 2b, c).

### Single and continuous administration of TMP-suppressed CCI-induced activation of MMP-9 and MMP-2 in vivo

**Fig. 3:**
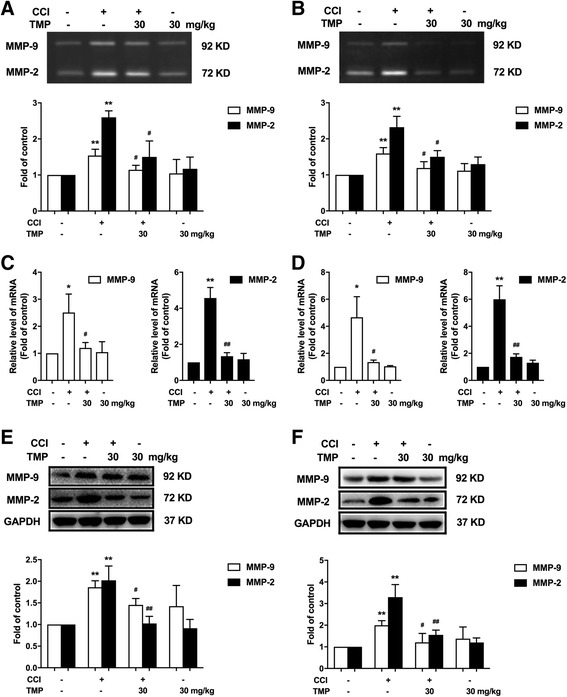
Single and consecutive administration of TMP significantly suppressed CCI-induced activation of MMP-2/9 in vivo. **a** Single administration of TMP (30 mg/kg, i.p.) significantly suppressed CCI-induced activation of MMP-2 and MMP-9 in the spinal cords. **b** Consecutive administration of TMP (30 mg/kg, i.p.) for 5 days from 14 days after the CCI operation significantly attenuated CCI-induced activation of MMP-2 and MMP-9 in the spinal cord. The gelatin zymography samples (*n* = 6) were collected as described above. **c** Single administration of TMP (30 mg/kg, i.p.) significantly attenuated CCI-induced increase in the expression of MMP-2 and MMP-9. **d** Consecutive administration of TMP (30 mg/kg, i.p.) significantly attenuated CCI-induced increase in the expression of MMP-2 and MMP-9. The levels of MMP-2 and MMP-9 mRNA in the spinal cord were determined using real-time quantitative PCR. GAPDH was used as an invariant control. **e** Single administration of TMP (30 mg/kg, i.p.) significantly attenuated CCI-induced increase in the expression of MMP-2 and MMP-9. **f** Consecutive administration of TMP (30 mg/kg, i.p.) significantly attenuated CCI-induced increase in the expression of MMP-2 and MMP-9. The lumbar spines (L1–L6) were collected and analyzed using western blot. A significant difference was at *P < 0.05, **P < 0.01 vs. control; ^#^ *P* < 0.05, ^##^ *P* < 0.01 vs. CCI group

To investigate the effects of TMP on MMP-9 and MMP-2 in vivo, single dosages of TMP were given intraperitoneally to CCI rats. Gelatin zymography results showed that TMP (30 mg/kg, i.p.) could inhibit the activity of MMP-9 and MMP-2 in the spinal cord (Fig. 3a). Furthermore, the action of TMP was studied by continuous administration for 5 days from 14 days after the CCI surgery. Consecutive administration of TMP (30 mg/kg, i.p.) for 5 days significantly inhibited the activity of MMP-9 and MMP-2 in the spinal cords from CCI-treated rats (Fig. 3b). The mRNA expression of the MMP-9 and MMP-2 were increased in CCI-treated rats (Fig. 3c, d). Compared with the CCI-treated rats, single and continuous administration of TMP (30 mg/kg, i.p.) significantly reduced these effects (Fig. 3c, d). Furthermore, single and continuous administration of TMP (30 mg/kg, i.p.) could significantly reduce CCI-induced protein expression of MMP-9 and MMP-2 in the spinal cord (Fig. 3e, f).

### TMP selectively inhibited CCI-induced JNK phosphorylation in astrocytes but not p38 or ERK

**Fig. 4:**
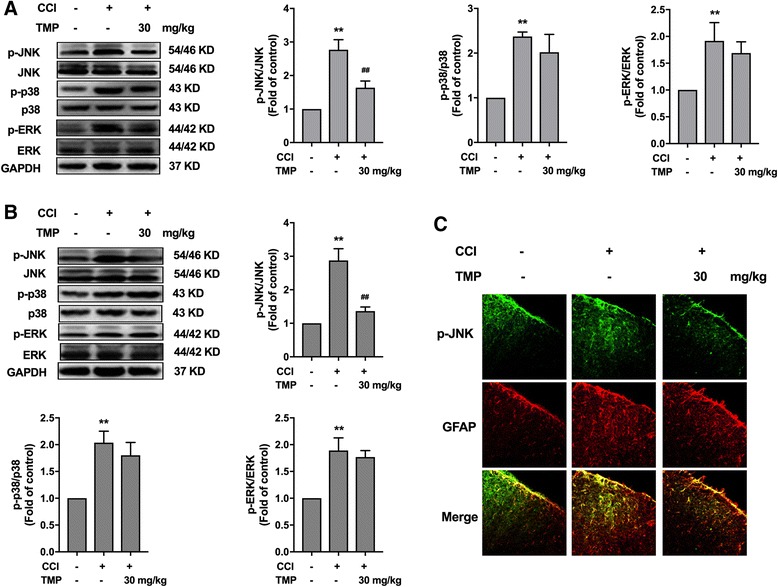
TMP selectively inhibited CCI-induced phosphorylation of JNK mitogen-activated protein kinase in the spinal cord. **a** Single administration of TMP (30 mg/kg, i.p.) inhibited CCI-induced phosphorylation of JNK after CCI operation, but not p38 or ERK. Representative bands and a data summary (*n* = 6) are shown. **b** Consecutive administration of TMP (30 mg/kg, i.p.) for 5 days from 14 days after CCI operation inhibited CCI-induced phosphorylation of JNK, but not p38 or ERK. Data summary (*n* = 6) is shown. Two-way ANOVA revealed a significant difference at *P < 0.05 and **P < 0.01 vs. control; and ^#^ *P* < 0.05 and ^##^ *P* < 0.01 vs. CCI group (Bonferroni post hoc tests). **c** Confocal images and immunofluorescence analysis data showing the effect of TMP (30 mg/kg, i.p.) on the expression of GFAP and p-JNK in the spinal cord. Quantification of immunofluorescence is represented as the mean fluorescence pixels in the superficial dorsal horns (*n* = 3, five images per animal). TMP (30 mg/kg, i.p.) was consecutively administered daily from the 14th day to the 18th day after CCI operation. The lumbar spines (L1–L6) were collected and analyzed 4 h after the last drug administration. A significant difference was at *P < 0.05, **P < 0.01 vs. control; ^#^ *P* < 0.05, ^##^ *P* < 0.01 vs. CCI group

Accumulating evidence shows that all three MAPK pathways contribute to pain sensitization after tissue and nerve injury via distinct molecular and cellular mechanisms. Because activation of MAPKs under different persistent pain conditions results in the induction and maintenance of pain hypersensitivity, we tested the effect of TMP on the MAPK family using western blotting. On the 14th day after the CCI operation, a single and continuous administration of TMP (30 mg/kg, i.p.) could significantly reduce CCI-induced phosphorylation of JNK in the spinal cord but not p38 or ERK (Fig. 4a, b). Immunofluorescence of GFAP and p-JNK in the dorsal horn showed a significant inhibition of TMP on CCI-induced activation of astrocytes (Fig. 4c).

### TMP significantly inhibited CCI-induced IL-1β cleavage, PKCγ phosphorylation, and NR1 phosphorylation

**Fig. 5:**
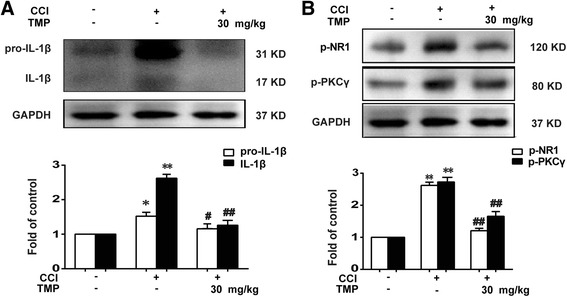
TMP significantly attenuated CCI-induced neuron cell activation and IL-1β production in spinal cords. **a** Single administration of TMP significantly inhibited the cleavage of IL-1β. TMP (30 mg/kg, i.p.) was administered on the 14th day after CCI operation. **b** Single administration of TMP inhibited CCI-induced phosphorylation of protein kinase C (PKC) γ and *N*-methyl-D-aspartate receptor (NR) 1. TMP (30 mg/kg, i.p.) was administered on the 14th day after CCI operation. The western blot samples (*n* = 6) were collected at 4 h after TMP administration. A significant difference was at *P < 0.05, **P < 0.01 vs. control; ^#^ *P* < 0.05, ^##^ *P* < 0.01 vs. CCI group

The above results proved that TMP significantly inhibited the activation of MMP-2/9 and attenuated chronic constrictive injury (CCI)-induced neuropathic pain. MMP-9 has the ability to cleave IL-1β, which is essential for pain generation. Here, we reported that a single and continuous administration of TMP (30 mg/kg, i.p.) blocked the production of maturated IL-1β induced by CCI (Fig. 5a).

IL-1β was expressed in DRG neurons and some satellite cells. We then evaluated the influence of TMP on neuronal activation induced by the CCI operation. TMP dramatically decreased CCI-induced phosphorylation of both PKCγ and NR1 by single administration of TMP (30 mg/kg, i.p.) (Fig. 5b).

### TMP significantly inhibited CCI-induced TAK1 phosphorylation; the TAK1 and JNK inhibitors can mimic the effect of TMP

**Fig. 6:**
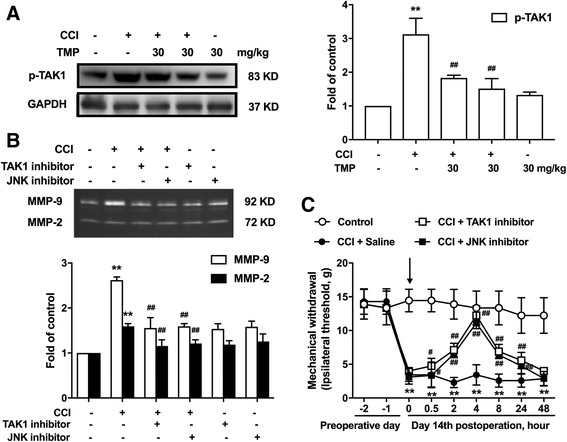
TMP attenuated the level of pTAK1, which could be mimicked by TAK1 and JNK inhibitors. **a** Single administration of TMP (30 kg/mg, i.p) after CCI operation 14 days significantly suppressed CCI-induced TAK1 phosphorylation in the spinal cord. Consecutive administration of TMP (30 kg/mg, i.p) for 5 days from 14 days after CCI operation significantly attenuated CCI-induced TAK1 phosphorylation. The western blot samples (*n* = 6) were collected as described above. **b** Administration of TAK1 inhibitor (5 μg/10 μl, i.t.) and JNK inhibitor (5 μg/10 μl, i.t.) significantly inhibited CCI-induced MMP-2 and MMP-9. **c** Administration of JNK inhibitor and TAK1 inhibitor inhibited CCI-induced mechanical allodynia. A significant difference was at *P < 0.05, **P < 0.01 vs. control; ^#^ *P* < 0.05, ^##^ *P* < 0.01 vs. CCI group

In the spinal cord, TAK1-induced activation of astrocytes is crucial for mechanical hypersensitivity after peripheral nerve injury. After nerve injury, TAK1 was increased in hyperactive astrocytes but not in neurons or microglia [16]. Here, we demonstrated that TMP (30 mg/kg, i.p.) could inhibit the activation of TAK1 phosphorylation in the spinal cords of rats in vivo (Fig. 6a). Furthermore, continuous administration of TMP (30 mg/kg, i.p.) for 5 days significantly inhibited the activity of TAK1 phosphorylation in the spinal cords of CCI-treated rats in vivo (Fig. 6a).

Furthermore, we found that JNK inhibitor and TAK1 inhibitor could suppress CCI-induced MMP-2 and MMP-9 (Fig. 6b). Administration of JNK and TAK1 inhibitor attenuated the maintenance CCI-induced neuropathic pain for the mechanical hyperalgesia thresholds, respectively (Fig. 6c).

### TMP inhibits the allodynia induced by IL-1β in vivo

**Fig. 7:**
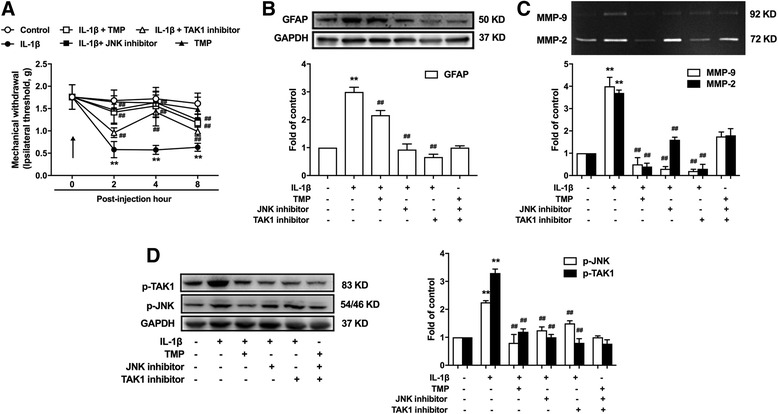
TMP inhibits IL-1β-induced TAK-1, JNK phosphorylation, and GFAP and MMP-2/9 activation of astrocytes in mice. **a** TMP (30 mg/kg, i.p.), the TAK1 inhibitor 5Z-7-oxozeaenol (5 μg/10 ul, i.t.) and the JNK inhibitor SP600125 (5 μg/10 ul, i.t.) attenuated the hyperalgia induced by intrathecal injection of 10 μl of IL-1β (20 ng/ml). **b** TMP, 5Z-7-oxozeaenol and SP600125 suppressed the higher expression levels of GFAP after the injection of IL-1β. **c** TMP, 5Z-7-oxozeaenol, and SP600125 inhibited the activation of MMP-2 and MMP-9 after intrathecal injection of 10 μl of IL-1β (20 ng/ml i.t.). **d** TMP, 5Z-7-oxozeaenol, and SP600125 inhibited the increased phosphorylation of JNK and TAK1 induced by IL-1β. A significant difference was at *P < 0.05, **P < 0.01 vs. control; ^#^ *P* < 0.05, ^##^ *P* < 0.01 vs. IL-1β group

The above results suggested that TMP, through TAK1/JNK, may inhibit the astrocytes activation induced by proinfammatory factors. We further investigated the effects of TMP on the allodynia and astrocytes activation induced by IL-1β. Injection of 10 μl of IL-1β (20 ng/ml, i.t.) into the spinal cord induced hyperalgia with a significant decrease in mechanical withdrawal. Treatment with TMP (30 mg/kg, i.p.) at 30 min before IL-1β injection attenuated the hyperalgia. Similarly, the TAK1 inhibitor 5Z-7-oxozeaenol (5 μg/10 ul, i.t., at 30 min before IL-1β injection) and the JNK inhibitor SP600125 (5 μg/10 ul, i.t., at 30 min before IL-1β injection) mimicked the ameliorative effects of TMP (Fig. 7a). Moreover, TMP, 5Z-7-oxozeaenol, and SP600125 suppressed the higher expression levels of GFAP after the injection of IL-1β (Fig. 7b). In addition, they could inhibit the increased MMP-2/9 and phosphorylation of JNK and TAK1 induced by IL-1β (Fig. 7c, d).

### TMP inhibits IL-1β-induced TAK-1 and JNK phosphorylation of astrocytes in vitro

To investigate the in vitro effects of TMP on proinflammatory factor-induced astrocyte activation, we used the immortalized murine astrocyte cell line C8, a clonal glial cell line derived from a rat glial tumor.

**Fig. 8:**
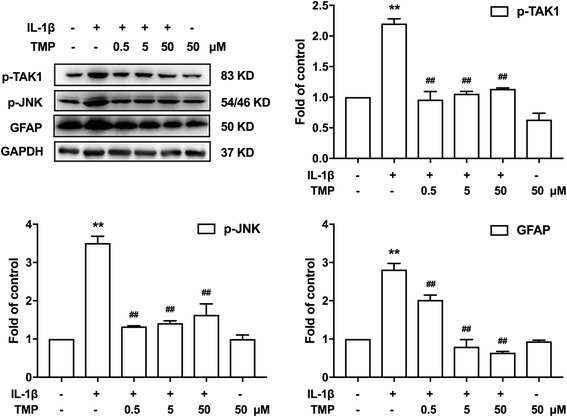
TMP inhibits IL-1β-induced TAK-1, JNK phosphorylation, and GFAP activation of astrocytes in vitro. Before the treatment of IL-1β (20 ng/ml), astrocyte cell line C8 were pretreated with different doses (0.5, 5, and 50 μM) of TMP for 30 min. TMP significantly reduced TAK-1, JNK phosphorylation, and GFAP. A significant difference was at *P < 0.05, **P < 0.01 vs. control; ^#^ *P* < 0.05, ^##^ *P* < 0.01 vs. IL-1β group

**Fig. 9:**
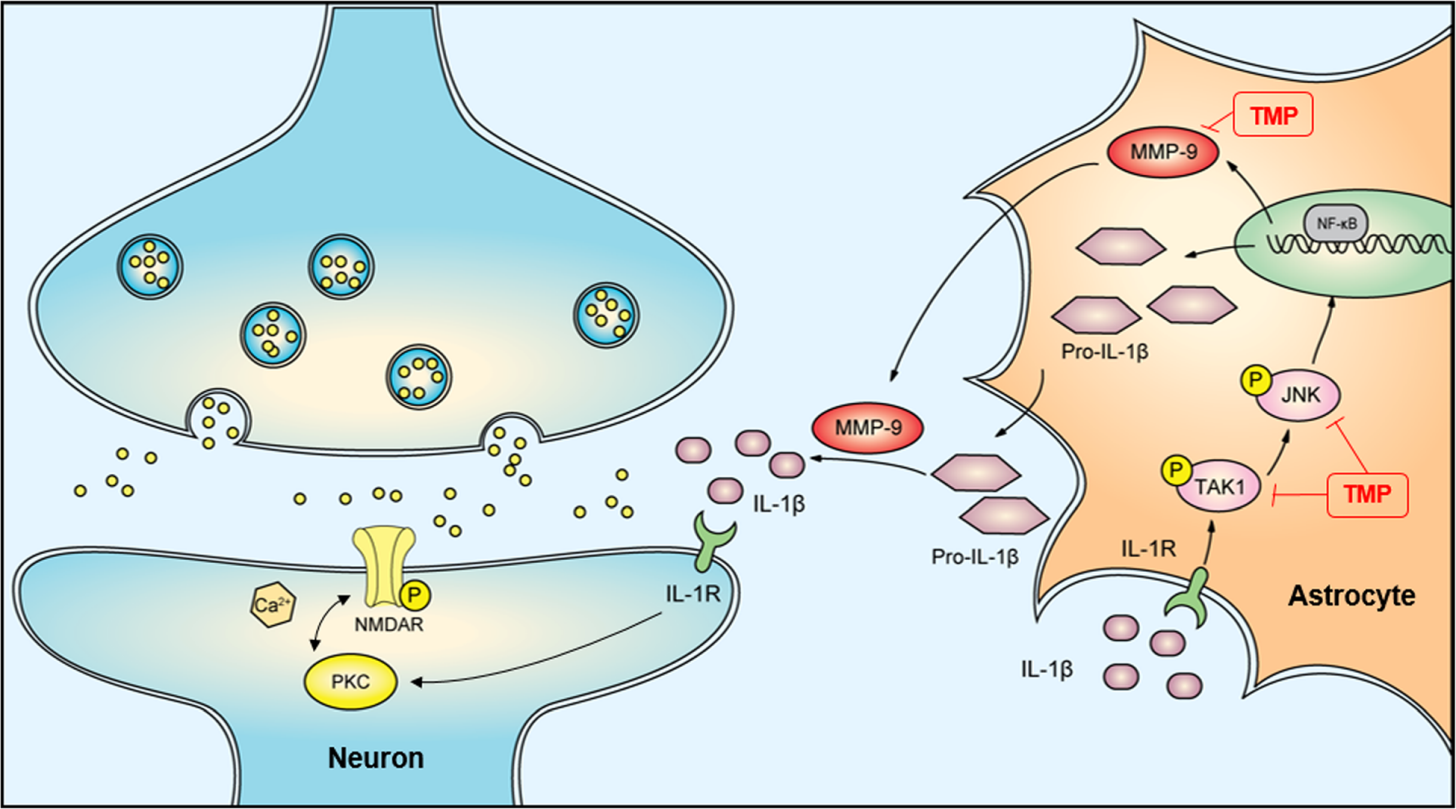
Schematic indicating the suppression of JNK-MMP2/9 in astrocytes to alleviate neuropathic pain by TMP. After CCI surgery, several pathways including the release of IL-1β, and calcium influx have been identified to signal the activation of astrocytes and neurons, leading to the neuroinflammation and central sensitization. TMP inhibited the MMP-2/9 activation, the maturation of IL-1β, and the activation of PKC and NMDA. The inhibition of TAK1-JNK-MMP2/9 was a critical mechanism for analgesic effect of TMP in astrocytes

Before the treatment with IL-1β (20 ng/ml), C8 cells were pretreated with different doses (0.5, 5, and 50 μM) of TMP for 30 min. We then analyzed the C8 cells after IL-1β exposure. The group that was pre-administered with TMP (0.5, 5, and 50 μM) significantly reduced TAK1 and JNK phosphorylation (Fig. 8). Based on the obtained results, the mechanisms of action and the underlying signaling pathways of TMP were proposed in Fig. 9.

## Discussion

In this study, our major findings included the following: (1) CCI-induced increases in MMP-2/9 in astrocytes may be mediated by JNK, (2) TMP significantly attenuated the maintenance of CCI-induced mechanical allodynia, (3) TMP inhibited CCI-induced spinal astrocyte activation, (4) TMP significantly suppressed the expression of MMP-2/9, and (5) TMP selectively suppressed phosphorylation of JNK via TAK1 signaling pathway, but had no effects on ERK and p38.

Activated astrocytes and mediated neuroinflammation are critical targets for neuropathic pain [17]. Spinal microglial activation in both dorsal and ventral horns peak 1 week after injury followed by a slow decline over several weeks, and astrocytes play an important role in the maintenance of neuropathic pain [18]. Astrocyte reactions after nerve injury, arthritis, and tumor growth are more persistent than microglial reactions, and they display a better correlation with chronic pain behaviors [7]. Studies report that the inhibition of activation of astrocytes could effectively attenuate neuropathic pain [18]. Our data show that either a single dose or continuous administration of TMP could significantly and dosage dependently attenuate CCI-induced neuropathic pain (Fig. 1). We also demonstrated that TMP significantly inhibited the upregulation of GFAP expression (Fig. 2). These data are consistent with previous results showing that astrocyte reactions are associated with the maintenance of pain [18].

Previous studies have shown that MMP-9 and MMP-2 have been considered key molecules for the onset and maintenance of neuropathic pain inhibition of astrocytes activation and could effectively attenuate neuropathic pain [18]. It has also been shown that MMP-2/9 contributes to the cleavage of IL-1β [10]. Then, increased IL-1β facilitates PKCγ phosphorylation through IL-1β receptors, leading to enhancement of NMDA receptor activity by NR1 subunit phosphorylation [19]. Activation of NMDA receptors subsequently induces Ca^2+^ influx and activates downstream signal cascade such as CaMKII [20]. These proteins can phosphorylate downstream molecules (e.g., PKCγ), which in turn lead to further activation of the NMDA receptor and contribution to central sensitization [21]. Our results have shown that TMP treatment significantly reduced the activity of MMP-2/9 in vivo induced by CCI or IL-1β (Figs. 4 and 7). We have also shown that the TMP-inhibiting activity of MMP-2 is more intense than that of MMP-9 (Fig. 3). Moreover, our data indicate that CCI-induced increases of IL-1β in the spinal cord were significantly inhibited by TMP (Fig. 5a), which were reconciled with the data in Fig. 7. In addition, the phosphorylation level of NR1 and PKC could also be suppressed by TMP (Fig. 5b, c). Taken together, our results suggest that inhibition of MMP-2/9 and IL-1β and that following the NR1 and PKC signal cascades could attenuate neuropathic pain.

Our present study and previous reports show pivotal roles of MMP-2/9 in pain-producing molecular signals not only in neurons [10] but also in glial cells in chronic pain states [12]. We further explored the detailed mechanism of TMP in inhibiting MMP-2/9 in astrocytes. We focused on the mitogen-activated protein kinase (MAPK) families. MAPK families are important for regulating neural plasticity and inflammatory responses and play essential roles in chronic pain [22]. The MAPK family has three major members including extracellular signal-regulated kinase (ERK), p38, and c-Jun N-terminal kinase (JNK) [23, 24]. Nerve injury or spinal cord injury induces a profound activation of MAPKs in the spinal cord [22]. Interestingly, MAPKs show a selective distribution by cell type. It is generally believed that activation of ERK occurs mainly in neurons, activation of p38 occurs mainly in microglia, and activation of c-Jun N-terminal kinase (JNK) occurs mainly in astrocytes. Our data showed that TMP significantly inhibits activation of JNK in spinal cord astrocytes and has no effect on ERK and p38 phosphorylation according to the results of a western blot, which was reconciled with immunofluorescence results (Fig. 4). To further confirm the direct suppressive effects of TMP on JNK, we repeated the experiments in cultured astrocytes. TMP still inhibited IL-1β-induced astrocyte activation, JNK phosphorylation, and the expression of MMP-2/9. Moreover, JNK inhibitor improved CCI-induced neuropathic pain (Fig. 6c) and inhibited MMP-2/9 levels (Fig. 6b).

We focused on the interactions between transforming growth factor-activated kinase 1 (TAK1) and TMP. We attended to the following aspects. First, TAK1 is a member of the MAPKKK family [25] and an upstream regulator of JNK [26]. Second, previous studies have already shown that TAK1 was mainly located in hyperactive astrocytes in the spinal cord after nerve injury [16]. Third, TAK1 was increased after nerve injury, and TAK1 inhibitor AS-ODN suppressed the activation of JNK in spinal astrocytes [16]. Our data indicates that after the CCI operation, the phosphorylation level of TAK1 increases and that TMP could suppress TAK1 expression in vivo (Fig. 6a). Further, administration of TAK1 inhibitor could attenuate neuropathic pain (Fig. 6c) and inhibit the expression of MMP-2/9 (Fig. 6b).

Our data suggest that TMP selectively suppressed the JNK signal pathway to inhibit the activation of astrocytes and then attenuated neuropathic pain via downregulation of TAK1 phosphorylation. However, it must be mentioned that we could not exclude downstream signaling pathway suppression, such as CaMKII. Previous studies have demonstrated that TMP could inhibit the activation of the calcium/calmodulin/calmodulin-dependent protein kinase (Ca2+/CaM/CaMKII) pathway [27]. TMP may suppress CaMKII to attenuate neuropathic pain. The exact mechanisms involved with TMP and CaMKII require further study.

## Conclusions

In summary, TMP could improve the maintenance of chronic constrictive injury (CCI)-induced neuropathic pain. Our results demonstrate that TMP regulates MMP-2 and MMP-9 signaling pathway in astrocytes by diminishing the phosphorylation of TAK1 and JNK, which are also important to improving neuropathic pain. Therefore, blocking the TAK1/JNK/MMPs signaling cascade in astrocytes might provide a fruitful strategy for treating intractable neuropathic pain. Altogether, our studies suggest that TMP may be a potential drug candidate for neuropathic pain treatment.

## Additional file

Additional file 1:
**Figure S1.** Additional controls with TMP treatment alone did not affect the baseline levels of p-p38, p-JNK, p-ERK MAPK family, IL-1β, p-NR1, and p-PKCγ in vivo. **Figure S2.** TMP did not show a notable influence on the microglia marker IBA1. A significant difference was at **P* < 0.05, ***P* < 0.01 vs. control. **Figure S3.** TMP did not show a notable influence on the MMP-2/9 in DRG. A significant difference was at **P* < 0.05, ***P* < 0.01 vs. control. (DOCX 574 kb)

## Abbreviations

BSA: Bovine serum albumin
DMEM: Dulbecco’s modified Eagle’s medium
DMSO: Dimethyl sulfoxide
ERK: Extracellular-regulated protein kinases
GAPDH: Glyceraldehyde-3-phosphate dehydrogenase
GFAP: Glial fbrillary acidic protein
IL-1β: Interleukin-1β
JNK: c-Jun N-terminal kinase
MAPK: Mitogen-activated protein kinase
MMP-2/9: Matrix metalloproteinase-2/9
NMDA: *N*-methyl-D-aspartic acid
NR1: *N*-methyl-Daspartate receptor 1
PBS: Phosphate-buffered saline
PKC: Protein kinase C
SDS-PAGE: Sodium dodecyl sulfate polyacrylamide gel electrophoresis
TAK1: Transforming growth factor beta-activated kinase 1
TMP: Tetramethylpyrazine
