# Supplementary material for: Selective suppression of the JNK-MMP2/9 signal pathway by tetramethylpyrazine attenuates neuropathic pain in rats
Source: J Neuroinflammation. 2017 Aug 31;14:174. doi: 10.1186/s12974-017-0947-x (PMC5580313; doi:10.1186/s12974-017-0947-x)
Supplement: Additional file 1: — Figure S1. Additional controls with TMP treatment alone did not affect the baseline levels of p-p38, p-JNK, p-ERK MAPK family, IL-1β, p-NR1, and p-PKCγ in vivo. Figure S2. TMP did not show a notable influence on the microglia marker IBA1. A significant difference was at *P < 0.05, **P < 0.01 vs. control. Figure S3. TMP did not show a notable influence on the MMP-2/9 in DRG. A significant difference was at *P < 0.05, **P < 0.01 vs. control. (DOCX 574 kb) [file 12974_2017_947_MOESM1_ESM.docx]

**Selective suppression of the JNK-MMP2/9 signal pathway by tetramethylpyrazine attenuates neuropathic pain in rats**

Lai Jiang ^1†^, Cai-Long Pan^1†^, Chao-yu Wang^1^, Bing-Qian Liu^2^, Yuan Han^3^, Liang Hu^1^, Lei Liu^4^, Yang Yang^5^, Jun-Wei Qu^5^, Wen-Tao Liu^1*^

Lai Jiang: [jltiffany1990@163.com](mailto:jltiffany1990@163.com); Cai-Long Pan: [pancailong@njmu.edu.cn](mailto:pancailong@njmu.edu.cn); Chao-yu Wang: wcy_0414@yeah.net; Bing-Qian Liu: [13151370243@qq.com](mailto:13151370243@qq.com); Yuan Han: [374198873@qq.com](mailto:374198873@qq.com); Liang Hu: [lianghu@njmu.edu.cn](mailto:lianghu@njmu.edu.cn); Lei Liu: [fort0825@sina.com](mailto:fort0825@sina.com); Yang Yang: jszlyyyy@163.com; Jun-Wei Qu: [junweiqu79@163.com](mailto:junweiqu79@163.com);

^1^ Jiangsu Key Laboratory of Neurodegeneration, Department of Pharmacology, Nanjing Medical University, Nanjing 210029, China

^2^ Ophthalmology, the First Affiliated Hospital with Nanjing Medical University, Nanjing 210029, China

^3^ Jiangsu Province Key Laboratory of Anesthesiology, Xuzhou Medical College, Xuzhou 221000, China

^4^ Department of pain, Shandong Qianfoshan Hospital, Shandong 250014, China

^5^ Department of Gynecologic Oncology, Jiangsu Cancer Hospital, Jiangsu Institute of Cancer Research, Nanjing Medical University Affiliated Cancer Hospital, Nanjing, Jiangsu 210009, China

^†^ Authors equally contribute to this work

*Addressed correspondence to Wen-Tao Liu, Jiangsu Key Laboratory of Neurodegeneration, Nanjing Medical University, 140 Han-Zhong Road, Nanjing 210029, China

Email: painresearch@njmu.edu.cn

Tel: +86-25-86862127; Fax: +86-25-86862127

**Figure S1**

**
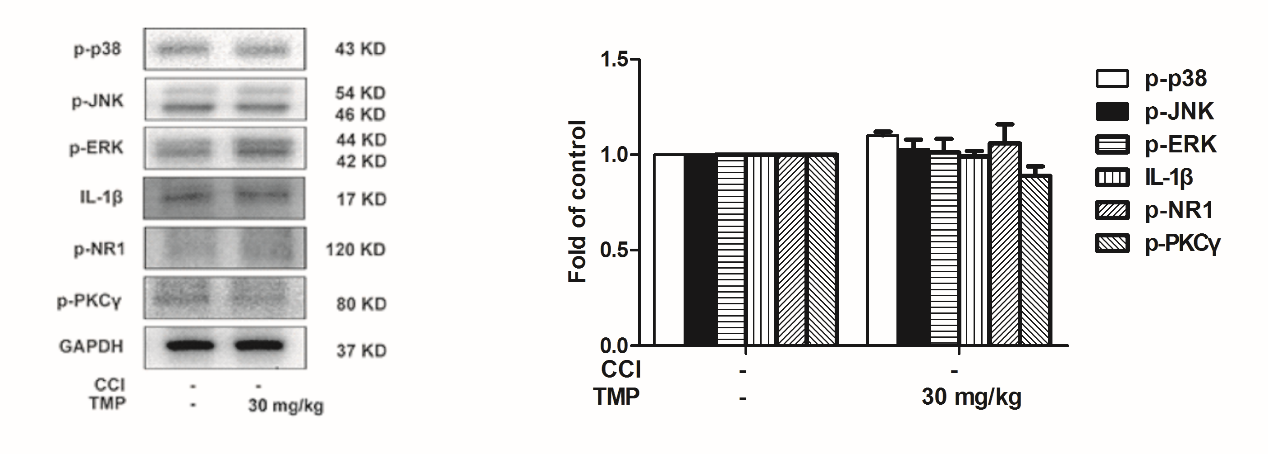
**

**Figure S1 S1** Additional controls with TMP treatment alone did not affect the baseline levels of p-p38, p-JNK, p-ERK MAPK family, IL-1β, p-NR1 and p-PKCγ in vivo.

**Figure S2**


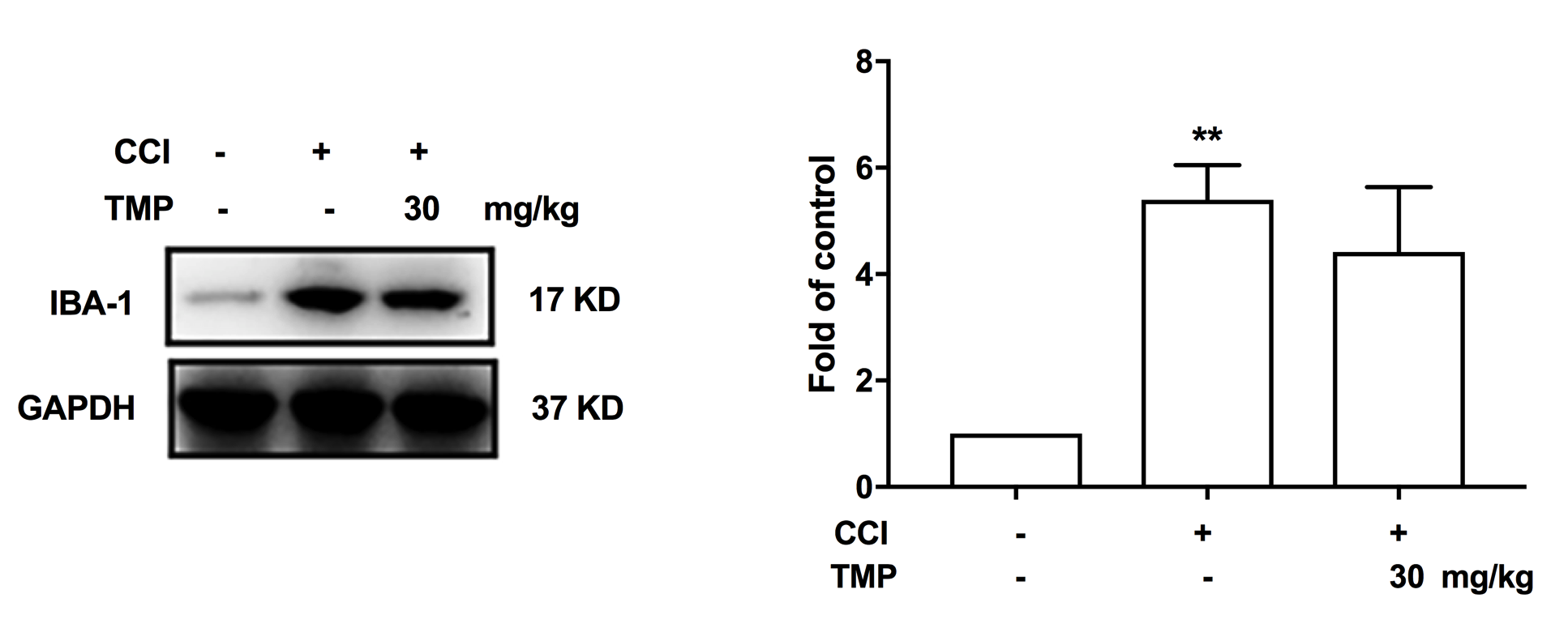


**Figure S1 S2** TMP did not show a notable influence on the microglia marker IBA1. A significant difference was at *P < 0.05, **P < 0.01 vs. control.

**Figure S3**


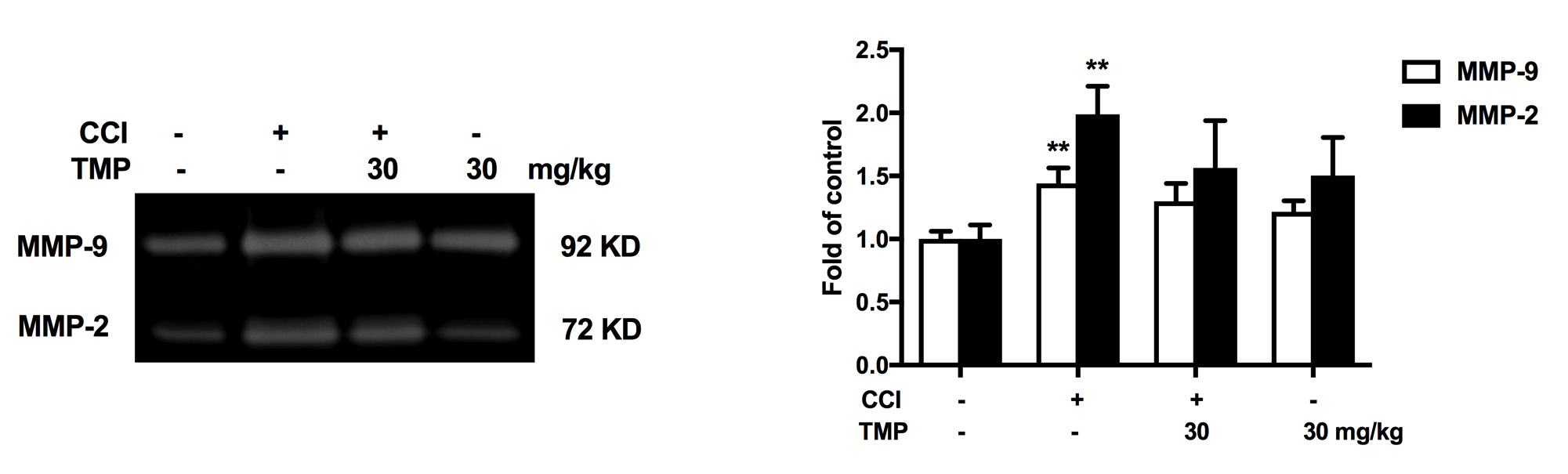


**Fig. S3** TMP did not show a notable influence on the MMP-2/9 in DRG. A significant difference was at *P < 0.05, **P < 0.01 vs. control.
